# In vitro propagation and genetic fidelity study of plant regenerated from inverted hypocotyl explants of eggplant (*Solanum melongena* L.) cv. Arka Shirish

**DOI:** 10.1007/s13205-012-0068-2

**Published:** 2012-06-06

**Authors:** N. Padma Mallaya, G. A. Ravishankar

**Affiliations:** Plant Cell Biotechnology Department, Central Food Technological Research Institute (CSIR), Mysore, 570 020 India

**Keywords:** *Solanummelongena* cv. Arka Shirish, Hypocotyl, RAPD, Shoot buds, Thidiazuron, Inverted

## Abstract

Genetic variation due to somaclonal variation in micropropagated plants is a beneficial phenomenon for crop improvement. Genetic integrity of the plants derived through micropropagation becomes crucial if genetic transformation studies have to be carried out. Somaclonal variation in tissue culture is a common phenomenon which makes it mandatory to check for genetic stability of plants. Hypocotyl explants of *Solanummelongena* L. cv. Arka Shirish inoculated with inverted polarity in MS media supplemented with 0.5 mg L^−1^ thidiazuron (TDZ) gave maximum number of shoot buds. Elongation of the shoot buds was achieved on MS medium supplemented with 0.5 mg L^−1^ 2, 3, 5-triiodobenzoic acid (TIBA) and 0.1 mg L^−1^ gibberellic acid (GA_3_). The elongated shoots were rooted in MS with 1 mg L^−1^ indole-3-butyric acid (IBA), and the rooted plants were hardened in the greenhouse. Morphological characteristics were similar in both seed-propagated and micropropagated plants. Random amplified polymorphic DNA analysis carried out with 10 primers for genetic stability studies of the regenerated plants generated 96 scorable bands with a total of 1,056 bands for the primers. Comparison of the bands with the mother plant revealed the monomorphic nature and true-to-type clones. The above regeneration protocol will be useful for micropropagation and genetic transformation studies of *S.melongena* L. cv. Arka Shirish.

## Introduction

Eggplant (*Solanummelongena* L*.*), a member of the Solanaceae family, is an economically important vegetable crop of Indian origin. Eggplant can be consumed raw, boiled, stuffed or made into soups or pickles (Asaolu and Asaolu 2002). Commonly known as brinjal in India, it is a good source of vitamins and minerals (Singh and Kumar 2006). Tissue extracts of eggplant can be used in the treatment of asthma, bronchitis, cholera and dysuria. Fruits and leaves of eggplant can be used for lowering blood cholesterol and can be given to diabetics and obese patients as it is low in calories and high in potassium (Kashyap et al. 2003; Singh and Kumar 2006; Rajam and Kumar 2007). It is mostly cultivated in tropical and temperate regions of the world. The three main varieties of eggplant include egg-shaped (*S*. *melongena* var. *esculentum*), long and slender in shape (*S*. *melongena* var. *serpentium*) and dwarf types (*S*. *melongena* var. *depressum*) (Rajam and Kumar 2007).

In eggplant, various protocols on in vitro regeneration have been carried out using various auxins and cytokinins either alone (Gleddie et al. 1983; Magioli et al. 1998; Mukherjee et al. 1991) or in combinations (Matsuoka and Hinata 1979) using various explants. Two different cytokinin combinations have been used for regeneration of eggplant from roots (Franklin et al. 2004). In spite of several protocols for regeneration being reported in eggplant, the regeneration efficiency has been shown to be influenced by explant type, genotype and also the morphogenetic response varying within the same explant (Sharma and Rajam 1995). The advent of new biotechnological approaches has opened up newer areas for eggplant micropropagation and genetic improvement. Micropropagation is the prerequisite for *Agrobacterium*-mediated genetic transformation studies in any plant, and true-to-type clonal fidelity is a must for micropropagation of any crop plant. Plant cell culture results in high frequency of variation in regenerated plants (Larkin and Scowcroft 1981). Owing to this variation, the resulting plant may not possess the same properties as that of the parent plant. These somaclonal variations can be detected through morphological, physiological/biochemical and molecular techniques (Bairu et al. 2011). Of these, molecular techniques are superior to morphological and biochemical techniques.

Various types of DNA-based molecular markers (RAPD, RFLP, ISSR, AFLP) have been used in applications in plant genetics and tissue culture studies (Sharma et al. 2007; Devarumath et al. 2002). Random amplified polymorphic DNA marker is often used in genetic variation studies in tissue-culture-derived plants when compared with restriction fragment length polymorphism (RFLP) because of the less quantity of DNA required, ease of use, low cost, reliability, less time consuming, and does not require prior knowledge of the nucleotide sequence of the organism under study, with no radioactive probes and no expensive restriction enzymes involved (Williams et al. 1990).

This paper reports regeneration of *S.melongena* L. cv. Arka Shirish, green long variety of eggplant from hypocotyl explants as well as studying the morphological characters, and also analyzing the genetic stability using RAPD technique of the greenhouse-grown tissue-cultured plants.

## Materials and methods

### Germplasm and explants used

Seeds of *S.melongena* L. cv. Arka Shirish were obtained from the Indian Institute of Horticultural Research, Bangalore, India. Seeds were thoroughly washed in running tap water and then surface sterilized with 0.1 % mercuric chloride (HgCl_2_). Mercuric chloride treated seeds were rinsed two to three times in sterile distilled water and soaked in sterile distilled water overnight. They were placed aseptically in Petri dishes with sterile filter paper soaked in distilled water for germination. The germinated seeds (85–90 %) were inoculated into half-strength MS medium (Murashige and Skoog 1962) devoid of any growth regulators. Hypocotyls from 15-day-old in vitro germinated seedlings were used as explants. These were devoid of roots, cotyledonary leaves and apical meristem. These were cut aseptically into 1-cm-long pieces and inoculated in horizontal mode. The entire hypocotyls of 5–7 cm length were used as explant for inoculating in inverted and vertical polarity for organogenesis.

### Culture media and conditions for shoot bud induction, elongation and rooting

MS media supplemented with 2 % sucrose was used as basal medium. Hypocotyls excised from seedlings were inoculated into basal medium with varying thidiazuron (TDZ; Sigma, USA) concentration for shoot bud induction. This hormone was selected based on the previous report of Magioli et al. (1998). For hypocotyl explants inoculated in horizontal mode, 3 Petri plates with 25 explants per plate were used, whereas for vertical and inverted mode of inoculation, 1 explant was cultured per tube. The TDZ concentrations used were 0.5, 1 and 2 mg L^−1^. The elongation of the shoot buds was obtained on basal medium with various concentrations of 2,3,5-triiodobenzoic acid (TIBA; Sigma, USA) and gibberellic acid (GA_3_; Sigma, USA) either alone or in combination. The elongated shoots were rooted in rooting medium having MS salts, vitamins, 3 % sucrose and varying concentrations of indole-3-butyric acid (IBA; Sigma, USA). Phytagel (3 % w/v) was used as the gelling agent for hypocotyl explants, which were to be inoculated in inverted mode. The pH of all the media were adjusted to 5.7 ± 0.2 prior to autoclaving at 1.06 kg cm^−2^ at a temperature of 121 °C for 15 min, which were dispensed into respective glasswares. The inoculated cultures were incubated at 25 ± 2 °C light under 16/8 h of photoperiod with 25 μmol m^−2^ s^−1^ light intensity. Growth measurements and data were collected periodically and analyzed statistically.

### Hardening of the rooted shoots

The rooted plants were washed off their agar under running tap water and transferred to plastic cups having sand:compost mixture (1:2) in greenhouse. These cups were covered with polyethylene bags with punched holes. The plantlets were hardened for 60 days and then transferred to pots with farmyard manure.

For comparative studies, seed-propagated plants were also grown under the identical greenhouse conditions.

### Characteristics of seed-propagated and tissue-cultured greenhouse-grown plants

#### Morphological analysis

Morphological traits of mature plants both of seed-propagated and tissue-culture-derived means were recorded (Table 4).

#### Estimation of total chlorophyll content in leaves

Total chlorophyll was extracted from mature leaf samples of seed-propagated and tissue-cultured plants using acetone as described by Jayaraman (1996). One  gram of fresh leaves were minced and homogenized with 10 mL distilled water; from this, 500 μL was taken and made up to 5 mL with 80 % acetone. This was centrifuged at 10,000 rpm for 5 min. The supernatant so obtained was used to measure the optical density at 645 and 663 nm. The total chlorophyll content in leaves was calculated as described by Jayaraman (1996) and was expressed as milligrams per gram fresh weight.

### Fruit quality traits

The following biochemical parameters were analyzed for the fruits of seed-propagated and tissue-cultured plants.

#### Total phenolic content in fruits

Sample preparation and total phenolic content determination were done as described by Samee et al. (2006) with slight modification. Mature fruits of both seed-propagated and tissue-cultured plants were harvested from greenhouse. One gram of each fruit was extracted with 80 % ethanol and centrifuged at 8,000 rpm for 10 min. The supernatant so obtained was saved. The residue was re-extracted twice with 80 % ethanol. The supernatants were pooled and evaporated to dryness at room temperature, and the extract was diluted with 3 mL of distilled water. An aliquot of 100 μL of the extract was again diluted to 3 mL with distilled water. To this 0.5 mL of Folin–Ciocalteu reagent (1: 10 diluted) was added. After 3 min, 2 mL of 20 % sodium carbonate was added and vortexed. The absorbance was measured at 750 nm. The results were expressed as gallic acid equivalent in milligrams per 100 g fresh weight material.

#### Total carbohydrate content in fruits

The total carbohydrates were estimated using anthrone reagent as described by Sadasivam and Manickam (2008). Essentially, 100 mg of seed-propagated and tissue-cultured fruits were hydrolyzed with 2.5 N HCl in boiling water bath for 3 h and then cooled to room temperature. This was then neutralized with solid sodium carbonate until the effervescence ceased. The supernatant so obtained after centrifugation was used to determine the total carbohydrate content, which was expressed as gram per 100 g fresh weight.

#### Total protein in fruits

The crude protein was extracted from seed-propagated and tissue-cultured fruits using 0.1 M potassium phosphate buffer of pH 6.8 containing 10 mM ascorbic acid. The total protein content was analyzed using Lowry et al.’s (1951) method. The total protein content in fruit was expressed as gram per 100 g fresh weight.

#### Moisture content in fruits

The fruits were dried at 60–70 °C overnight in an oven, and the reduction in weight was calculated according to official method (AOCS 2003). The moisture content was expressed in percentage.

#### Mineral content analysis in fruits

Oven dried fruits were incinerated in a muffle furnace at 550 °C until ash. The ash so obtained was taken in aqua regia. Zinc, iron, potassium, magnesium and sodium were estimated by atomic absorption flame emission spectroscopy (Model AA-670IF; Shimadzu Corporation) with a graphite furnace attachment (Khan et al. 2011) after diluting with the respective acid. The minerals were quantified using reference standards. Potassium was analyzed using 2 % strontium chloride as the matrix modifier. They were expressed as milligrams per 100 g fresh weight.

### Genetic stability analysis using RAPD

Genomic DNA was isolated from fresh young leaves of ten micropropagated and seed-propagated plants grown in greenhouse using HiPure Plant genomic DNA extraction kit (Hi Media, India). Quantification of DNA was done based on spectrophotometric analysis. The samples were diluted to a concentration of 25 ng μL^−1^. A total of 30 primers (Sigma, St. Louis, Missouri) were used for RAPD analysis and out of which 10 were selected based on the reproducibility of the bands. RAPD amplifications were performed using 25 μL PCR mixture containing 25 ng of genomic DNA, 1× PCR buffer (Bangalore Genei, Bangalore, India), 200 μM dNTPs (Bangalore Genei, Bangalore, India), 1 U *Taq* DNA polymerase (Sigma) and 1 μM of each primer (Sigma, St.Louis, Missouri) in a thermal cycler (Eppendorf Mastercycler^®^ Hamburg Germany). The PCR conditions were 94 °C for 4 min, followed by 36 cycles of amplification with 1 min denaturation at 94 °C, 1 min annealing at 37 °C, and 1.5 min extension at 72 °C, and a final extension of 72 °C for 10 min. The PCR products were electrophoresed on 1.5 % agarose gel with 10 kb DNA marker (MBI Fermentas, Lithuania) and documented using Hero-Lab GmbH (Germany). RAPD analysis was repeated twice using each primer to establish reproducibility of the banding pattern of different DNA samples of *S. melongena*.

### Statistical and RAPD analysis

All the experiments were repeated thrice and the values were represented as mean ± standard error. The results were statistically analyzed using one-way ANOVA (Origin Pro 8) (Tables 1–4), and the mean differences were analyzed by Fisher LSD test at a probability level of *p* < 0.05. For RAPD analysis, consistent and well-resolved bands were scored manually as ‘1’ if present and ‘0’ if absent in the gel. To detect any genetic change, the results were compared with the seed-propagated plants as well as among the micropropagated plants.

**Table 1:** Shoot bud induction in hypocotyl explants of *S. melongena* L.

Explant orientation	TDZ (mg L^−1^)	No. of shoot buds/explant^#^
Horizontal	0.5	31 ± 1.9^d^
	1	37 ± 0.9^e^
	2	7 ± 0.3^b^
Vertical	0.5	1 ± 0.3^a^
	1	1 ± 0.6^a^
	2	1 ± 0.6^a^
Inverted	0.5	40 ± 1.5^f^
	1	31 ± 0.9^d^
	2	14 ± 0.6^c^

Data recorded after 30 days

*TDZ* thidiazuron

^#^Mean ± standard error of three replicates. Means followed by the same letter are not significantly different using Fisher LSD at *p* < 0.05

## Results and discussion

### Shoot multiplication, rooting and hardening

**Fig. 1:**
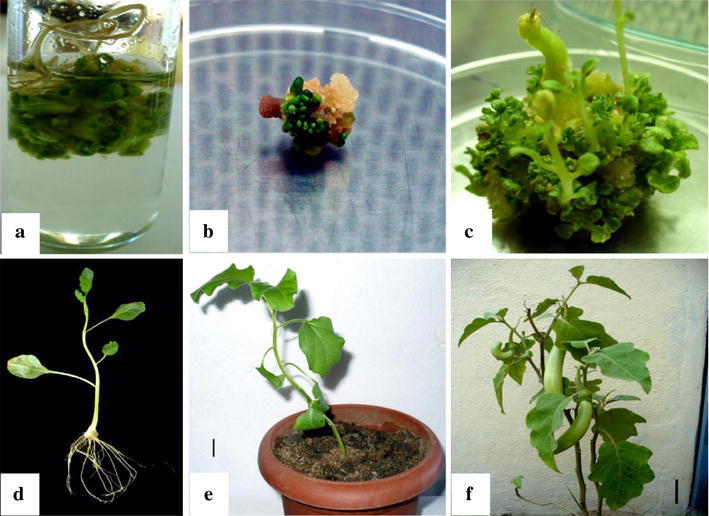
**a** Shoot buds from hypocotyl explant of *S. melongena* inoculated in inverted orientation in MS medium supplemented with 0.5 mg L^−1^ thidiazuron (TDZ). **b** Shoot buds from hypocotyl explants of *S. melongena* inoculated in horizontal direction in MS medium supplemented with 1 mg L^−1^ TDZ. **c** Shoot buds elongation from hypocotyl explants of *S. melongena* inoculated in inverted orientation in 0.5 mg L^−1^ 2,3,5-triiodobenzoic acid (TIBA) and 0.1 mg L^−1^ gibberellic acid (GA_3_). **d** Rooted plant of *S. melongena* in MS medium supplemented with 1 mg L^−1^ indole-3-butyric acid (IBA). **e** Hardened plant in greenhouse. *Scale* = 3.5 cm. **f** Hardened plant with fruit. *Scale* = 10.0 cm

All explants gave shoot buds within 30 days of inoculation in MS medium with TDZ. Among all, hypocotyl explants in inverted orientation gave the maximum number of shoot buds per explant (40 ± 1.5) in 0.5 mg L^−1^ TDZ (Table 1; Fig. 1a). The maximum number of shoot buds 37 ± 0.9 per explant was obtained in 1 mg L^−1^ TDZ concentration for hypocotyl in horizontal orientation (Table 1; Fig. 1b). The shoot buds elongated in 0.5 mg L^−1^ TIBA and 0.1 mg L^−1^ GA_3_ combination gave the maximum elongated shoots (19 ± 0.6 per explant) of shoot length 3.3 ± 0.2 cm per explant (Table 2; Fig. 1c) in 30 days of inoculation. No increase in the number of shoot buds was observed when the cultures were transferred from TDZ to MS basal salts supplemented with 0.5 mg L^−1^ TIBA and 0.1 mg L^−1^ GA_3_ and 2 % sucrose media.

**Table 2:** Elongation of shoot buds of *S. melongena* L.

Growth regulator MS + 2 % sucrose		No. of shoots buds elongated^#^	Shoot length (cm)^#^
TIBA (mg L^−1^)	GA_3_ (mg L^−1^)		
0	0	1 ± 0.3^a^	0.4 ± 0.1^a^
0	0.05	9 ± 1.5^bc^	1.2 ± 0.4^abc^
0	0.1	9 ± 0.3^bc^	2.0 ± 0.6^cdeg^
0	0.5	8 ± 1.5^b^	1.5 ± 0.4^bce^
0.1	0.1	10 ± 0.9^bcd^	2.5 ± 0.3^defg^
0.1	0.5	11 ± 1.2 ^cd^	2.4 ± 0.2^efg^
0.5	0.1	19 ± 0.6^e^	3.3 ± 0.2^fg^
0.5	0.5	13 ± 0.7^d^	2.7 ± 0.1^g^

Data recorded after 30 days

*TIBA* 2,3,5-triiodobenzoic acid, *GA*_*3*_ gibberellic acid

^#^Mean ± standard error of three replicates. In a column, means followed by the same letter are not significantly different using Fisher LSD at *p* < 0.05

The various explants of eggplant have been reported for the induction of organogenesis in different media combination, which include hypocotyls (Magioli et al. 1998; Matsuoka and Hinata 1979), leaf (Gleddie et al. 1983; Magioli et al. 1998; Mukherjee et al. 1991), roots (Franklin et al. 2004), cotyledons (Magioli et al. 1998), nodes (Magioli et al. 1998) and epicotyls (Magioli et al. 1998). The differences in response of the explants maybe due to the regeneration efficiency of the explants, which is influenced by explant type, genotype and also the morphogenetic response varying within the same explant (Sharma and Rajam 1995). Organogenesis from root explants reported by Franklin et al. (2004) showed decrease in organogenetic potential of the root explants with age. However, shoot buds via intervening callus stage were reported by them in TDZ along with BAP media for root explants.

In the present study, culturing of hypocotyl with inverted polarity gave maximum shoot buds than the hypocotyl inoculated in horizontal direction. This may be due to the new meristematic activity of the apical organogenic region which is in direct contact with the medium under the influence of growth regulators as reported by Kumar et al. (2005) in *Capsicumannuum* L. Radiolabelling of growth regulators in hypocotyl explants of *Pelargonium* × *hortorum* Bailey have shown that auxin transport increases when apical region of the explant is in contact with the medium (Murch and Saxena 2001).

Prolonged exposure to TDZ media is reported to result in short shoots, which often fail to develop roots (Magioli et al. 1998). We used combination of TIBA and GA_3_ for the elongation of shoot buds based on the observation of Cambecédes et al. (1991) in *Lonicera nitida* Wils. cv. ‘Maigriin’. They hypothesized that TIBA may cause inhibition of auxin transport to regeneration site as a result of which a balance between auxin and cytokinin was established, which helped in shoot regeneration of *L. nitida* Wils. cv. ‘Maigriin’ (Cambecédes et al. 1991).

The elongated shoots were rooted in 1 mg L^−1^ IBA after 30 days of inoculation (Table 3; Fig. 1d), which gave maximum number or roots 4 ± 0.7 per explant of root length 5.6 ± 1.1 cm per explant. The rooted plants were transferred to small plastic cups and covered with polyethylene cover with holes for hardening under greenhouse conditions. They were hardened under greenhouse conditions for 2 months (Fig. 1e) and later transferred to pots containing farmyard manure. About 80 % of the plants survived the hardening and grew into mature fruit bearing plants (Fig. 1f).

**Table 3:** Rooting of elongated shoots of *S. melongena* L.

IBA (mg L^−1^)	No. of roots	Shoot length (cm)^#^	Root length (cm)^#^
0.1	0 ± 0^a^	3.7 ± 0.9^b^	0 ± 0^a^
0.25	1 ± 0^b^	2.0 ± 0.7^a^	0.8 ± 0.4^a^
0.5	2 ± 0.6^b^	3.9 ± 0.2^bc^	5.8 ± 0.3^c^
1	4 ± 0.7^c^	4.9 ± 0.8^c^	5.6 ± 1.1^bc^

Data recorded after 30 days

*IBA* indole-3-butyric acid

^#^Mean ± standard error of three replicates. In a column, means followed by the same letter are not significantly different using Fisher LSD at *p* < 0.05

### Morphological characteristic and RAPD analysis of the greenhouse-grown plants

Somaclonal variation during in vitro propagation may arise from pre-existing variations, the type of explant used, the concentration and type of growth regulator in the medium, number and duration of subcultures, effect of stress, genotype and the method of propagation adopted. The chance of variation is more when plants are regenerated via an intermediate callus phase (Bairu et al. 2011). The level of synthetic plant growth regulators in the medium is also coupled with somaclonal variation (Martin et al. 2006).

In this study, we have regenerated plants from hypocotyl explants using TDZ, a synthetic plant growth regulator, and hence it becomes obligatory to check for the genetic stability of the regenerated plant before it could be used for *Agrobacterium*-mediated genetic transformation studies.

As part of preliminary analysis of the plant for somaclonal variation, the micropropagated plants were compared with the seed-propagated plants in terms of morphological characteristics. In our study, the morphological characters like fruiting and flowering pattern resembled the conventionally propagated plant (Table 4). Bhatia and Ashwath (2004) also observed no change in phenotypic characters (plant height, flowering peduncles, average fruit diameter, etc.) between the tissue-cultured and seed-propagated tomato (*Lycopersicon esculentum* Mill. cv. Red Coat). The total chlorophyll content and the fruit quality traits like the total proteins, total carbohydrates, mineral content, moisture content, total phenolic content in the tissue-cultured plants of *S.melongena*, and seed-propagated plant did not show variation (Table 4).

**Table 4:** Morphological and biochemical characteristics of seed-propagated and tissue-cultured grown plants under greenhouse condition

	Seed-propagated plant^#^	Tissue-cultured plant^#^
Morphological		
Stem: anthocyanin coloration	Absent	Absent
Leaf: length (cm)	22.3 ± 1.0^a^	24 ± 0.6^a^
Leaf: width (cm)	23.5 ± 0.9^a^	22 ± 1.2^a^
Leaf: spininess	Absent	Absent
Leaf: color of vein	Green	Green
Inflorescence: no. of flowers	1–3	1–3
Fruit: length (cm)	27.1 ± 0.5^a^	25.2 ± 1.7^a^
Fruit: length to diameter ratio	2.09	2.12
Fruit: color of skin at harvesting	Green	Green
Fruit: color of calyx	Green	Green
Fruiting pattern	Solitary	Solitary
Fruit weight (g)	150.5 ± 0.2^a^	148.5 ± 0.9^a^
Plant: height (cm)	72.5 ± 1.4^a^	69.5 ± 2.6^a^
Total chlorophyll content in leaves (mg g^−1^ FW)	1.2 ± 0.002^a^	1.2 ± 0.002^a^
Fruit quality traits		
Moisture content (%)	90.9 ± 0.1^a^	89.8 ± 0.7^a^
Total proteins (g/100 g FW)	0.9 ± 0.04^a^	1.0 ± 0.08^a^
Total carbohydrates (g/100 g FW)	8.0 ± 1.6^a^	8.2 ± 0.4^a^
Total phenolic content (mg GAE/100 g FW)	737.3 ± 28.9^a^	740.0 ± 52.9^a^
Iron (mg/100 g FW)	0.3 ± 0.06^a^	0.2 ± 0.05^a^
Zinc (mg/100 g FW)	0.2 ± 0.03^a^	0.2 ± 0.00^a^
Sodium (mg/100 g FW)	3.3 ± 0.15^a^	3.6 ± 0.03^a^
Potassium (mg/100 g FW)	344 ± 2.3^a^	341 ± 1.5^a^
Magnesium (mg/100 g FW)	13.7 ± 0.09^a^	14.6 ± 0.15^a^

*FW* fresh weight, *GAE* gallic acid equivalent

^#^Mean ± standard error of three replicates. In a row, means followed by the same letter are not significantly different using Fisher LSD at *p* < 0.05

It was seen that morphological analysis of the micropropagated plants did not show any variations, but these markers have the limitations of being dependent on environmental factors and do not represent the genetic constitution of the plant (Mandal et al. 2001). The regenerated plants were checked for their genetic stability using RAPD primers. Even though numerous protocols for eggplant micropropagation are available, somaclonal variation studies in eggplant are limited (Magioli and Mansur 2005; Collonnier et al. 2001). Only a few reports on molecular marker-based analysis of somaclonal variation is available in eggplant (Xing et al. 2010). Molecular markers have been used in eggplant for assessing genetic diversity and varietal differences, and in the construction of genetic linkage maps for the identification of useful agronomic traits (Collonnier et al. 2001; Kashyap et al. 2003). RAPD has been widely used in genetic variation studies in tissue-culture-derived plants as has been reported in *Silybummarianum* (L.) (Mahmood et al. 2010), Date Palm (Saker et al. 2000), and hop (*Humulus lupulus* L.) (Patzak 2003). Whereas in gerbera (*Gerberajamesonii* Bolus) (Bhatia et al. 2010), turmeric (Nayak et al. 2011) and *Zingiberrubens* (Mohanty et al. 2011) RAPD analysis showed absence of genetic variation.

**Fig. 2:**
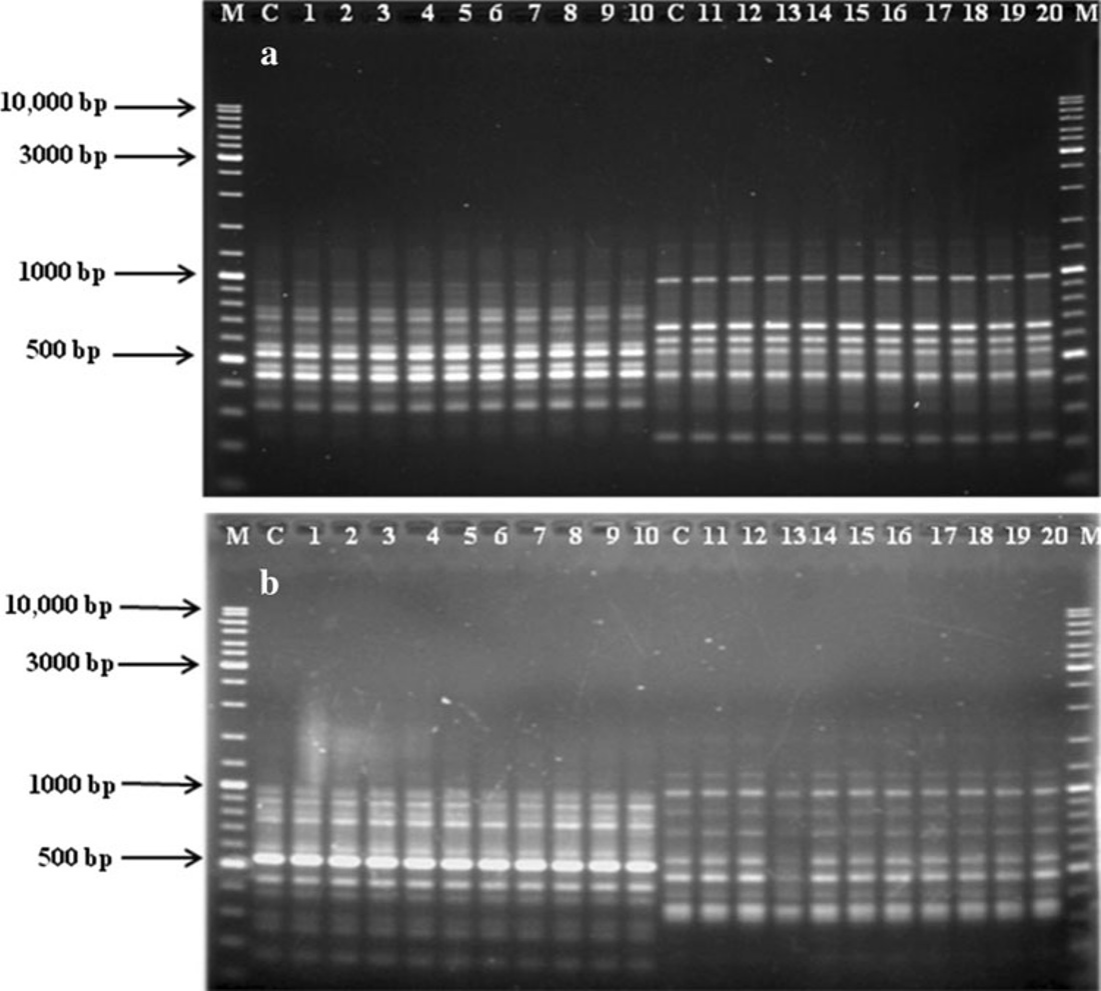
**a** RAPD profiles using the primers OPB7 [*lane C* control plant, *lanes 1–10* micropropagated plants, *M* 10 kb marker (Fermentas)] and OPA7 [*lane C* control plant, *lanes 11–20* micropropagated plants, *M* 10 kb marker (Fermentas)]. **b** RAPD profiles using the primers OPA14 [*lane C* control plant, *lanes 1–10* micropropagated plants, *M* 10 kb marker (Fermentas)] and OPD16 [*lane C* control plant, *lanes 11–20* micropropagated plants, *M* 10 kb marker (Fermentas)]

Of the 30 RAPD primers used for preliminary screening of the control plants, only ten gave clear and distinct scorable bands. These primers were further used for the analysis of the micropropagated plants. The 10 primers generated 96 scorable bands. The number of scorable bands varied from 6 (OPA-11) to 14 (OPA-06) with an average of 9.6 bands per primer. The size range for the bands varied from 175 to 1,800 bp (Table 5). A total number of 1,056 bands were generated (number of plants analyzed × number of bands obtained with RAPD primers analyzed). Comparison of the banding pattern between the micropropagated and seed-propagated plants revealed the absence of any polymorphic bands (Fig. 2).

**Table 5:** List of primers with respective sequence, number and size range of bands generated with the RAPD primers

Sl No.	Primer	Primer sequence 5′–3′	No. of scorable bands	Size range (bp)
1	OPA-06	GGTCCCTGAC	14	375–1,250
2	OPA-07	GAAACGGGTG	7	220–980
3	OPA-11	CAATCGCCGT	6	600–1,550
4	OPA-14	CTCGTGCTGG	11	175–1,000
5	OPB-07	GGTGACGCAG	12	320–980
6	OPC-06	GAACGGACTC	10	500–1,800
7	OPC-20	ACTTCGCCAC	9	600–1,400
8	OPD-16	AGGGCGTAAG	8	300–1,200
9	OPJ-04	CCGAACACGG	8	500–1,300
10	OPM-16	GTAACCAGCC	11	330–1,000
Total			96

In our studies, the crucial aspects, such as flowering, fruit setting and fruit characteristics, did not alter between seed-propagated and tissue-cultured plants. RAPD analysis of the micropropagated plants also showed genetic stability. Hence, it can be concluded that the micropropagation protocol developed in this study is suitable for micropropagation and in the genetic transformation studies of this economically important food value crop both in post-harvest and pre-harvest quality improvements.
